# Supplementary material for: Systemic lupus erythematosus favors the generation of IL-17 producing double negative T cells
Source: Nat Commun. 2020 Jun 5;11:2859. doi: 10.1038/s41467-020-16636-4 (PMC7275084; doi:10.1038/s41467-020-16636-4)
Supplement: Supplementary file 1 — Supplementary Information [file 41467_2020_16636_MOESM1_ESM.pdf]

Systemic lupus erythematosus milieu favors the generation of IL-17 producing double negative T cells

Li H, Tsokos G *et al.*

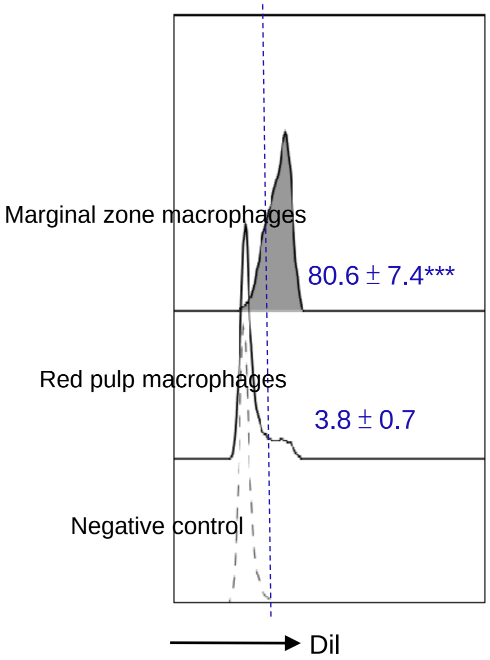

Supplementary Figure 1. Characterization of liposome delivery specificity in vivo.

B6.*lpr* mice were treated with DiI-liposome (100ug/mouse). Mice were euthanized 30 min after administration for analysis. Flow cytometry analysis of targeting specificity by examining DiI intensity in different splenic subset macrophages. Red pulp macrophages: $CD11b^{+}CD68^{+}F4/80^{+}$, Marginal zone macrophages: $CD11b^{lo}F4/80^{-}I\text{-}A^{b-}SIGN\text{-}R1^{+}$, *n* = 3 mice per group. Data represent the mean ± SEM (***P < 0.005 vs. control, two-tailed student's *t* test; *n* = 3 mice per group).

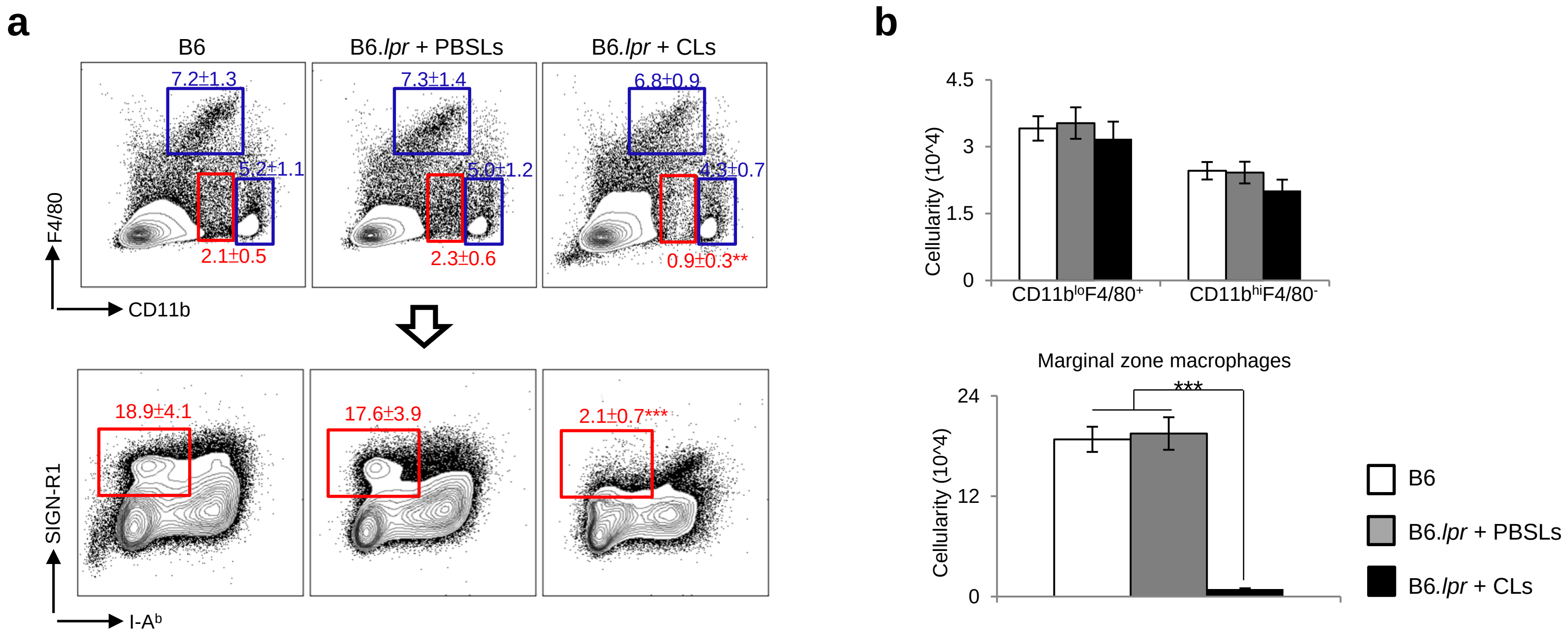

Supplementary Figure 2. Depletion of MZMs *in vivo* by CLs.

Age-matched female B6.*lpr* mice were treated with either PBS-loaded control liposomes (PBSLs) or clodronate liposomes (CLs, 100 ug/mouse) every other week for 2 months total starting at 10 weeks of age. Naïve B6 mice were used as control. (a) Flow cytometry quantitation of the percentage of MZMs (CD11b^{lo}F4/80^{-}I-A^{b-}SIGN-R1^{+}) in the spleens from indicated mice. Data represent the mean ± SEM. (b) Bar graphs show the cellularity of indicated populations in the spleens of indicated mice. (***P* < 0.01, ****P* < 0.005 vs. control, two-tailed student's *t* test; *n* = 5-6 mice per group for 2 independent experiments).

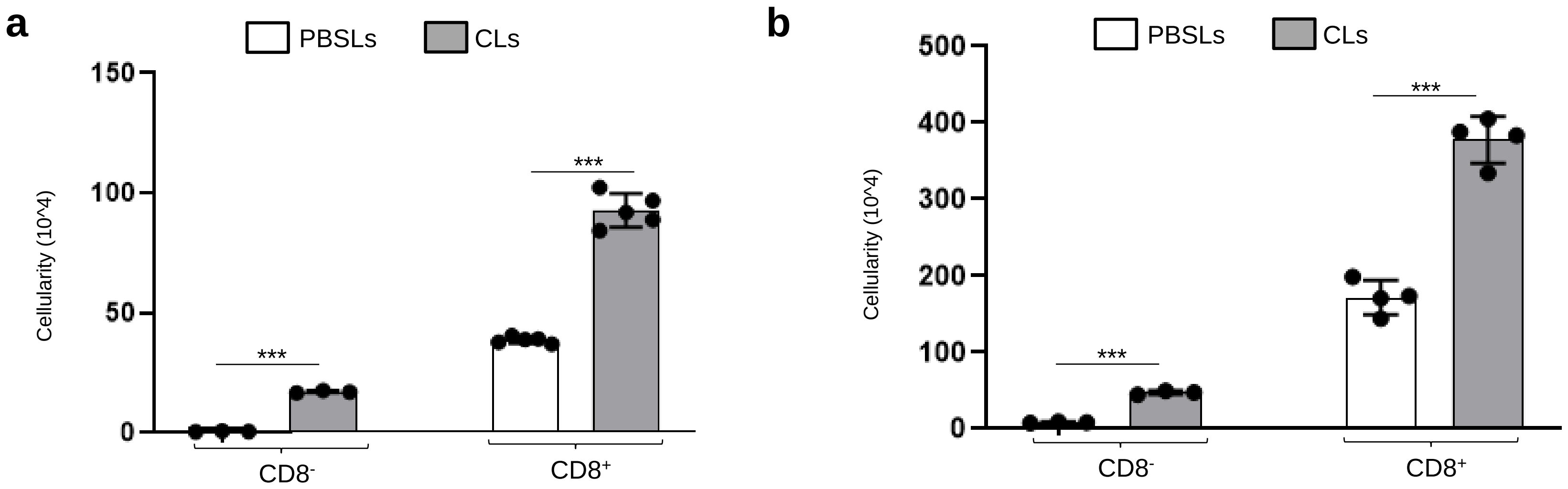

Supplementary Figure 3. Exposure to self-antigens derived from apoptotic cell debris expands DN like T cells from $CD8^+$ T cells.

(a) OT-I TCR Tg CD8 T cells from CD45.1 OT-I TCR Tg *Rag1*$^{-/-}$ B6 mice were transferred i.v. (5 x 10^6 per mouse) into B6 mice. Recipients were administered CLs or control liposomes, apoptotic thymocytes prepared from m-OVA Tg mice (2 x 10^7 per mouse), 12 hrs and 16 hrs later sequentially. After an additional 72 hrs, mice were sacrificed for analysis. Bar graphs show the cellularity of $CD8^+$ and $CD8^-$ OT-I T cells after transfer.

(b) OT-I TCR Tg CD8 T cells from CD45.1 OT-I TCR Tg *Rag1*$^{-/-}$ B6 mice were transferred i.v. (5 x 10^6 per mouse into m-OVA Tg B6 mice administered CLs or control liposome 4 hrs before and sacrificed after an additional 72 hrs. After an additional 72 hrs, mice were sacrificed for analysis. Bar graphs show the cellularity of $CD8^+$ and $CD8^-$ OT-I T cells after transfer.

Data represents the mean ± SEM, ****P* < 0.005 vs. PBSLs treated controls, two-tailed student's *t* test. *n* = 4-5 mice per group for 2 independent experiments.

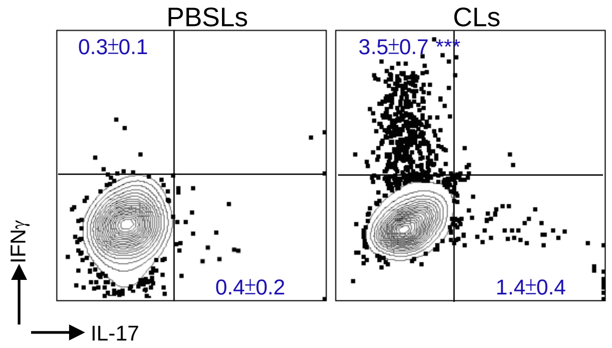

Supplementary Figure 4. Absence of MZMs drives the activation of transferred OT-II T cells in the presence of exogenous antigens derived from apoptotic thymocytes.

OT-II TCR Tg CD4 T cells from CD90.1 OT-I TCR Tg Rag1$^{-/-}$ B6 mice were transferred i.v. (5 x 10^6 per mouse) into B6 mice. Recipients were administered CLs or control liposome, apoptotic thymocytes prepared from m-OVA Tg mice (2 x 10^7 per mouse), 12 hrs and 16 hrs later sequentially. After an additional 72 hrs, mice were sacrificed for analysis. Flow cytometry quantitation of the percentage of IL-17^{+} and IFN-γ^{+} cells in transferred OT-II TCR Tg CD4 T cells. Data represent the mean ± SEM (****P* < 0.005 vs. control, two-tailed student's *t* test; *n* = 4-5 mice per group for 2 independent experiments).

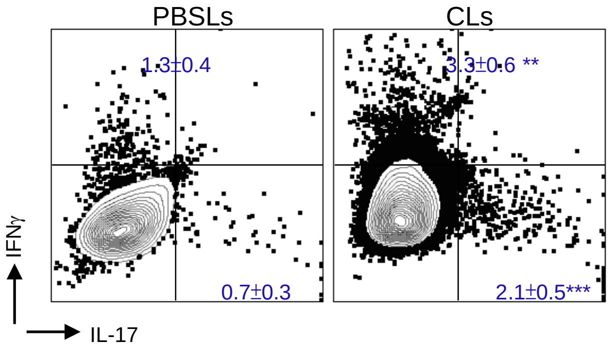

Supplementary Figure 5. Absence of MZMs drives the activation of transferred OT-II T cells in the presence of endogenous antigens.

OT-II TCR Tg CD4 T cells from CD90.1 OT-I TCR Tg Rag1$^{-/-}$ B6 mice were transferred i.v. (5 x 10^6 per mouse) into m-OVA Tg B6 mice administered CLs or control liposome 4 hrs before and sacrificed after an additional 72 hrs. Flow cytometry quantitation of the percentage of IL-17^{+} and IFN-γ^{+} cells in transferred OT-II TCR Tg CD4 T cells. Data represent the mean ± SEM (***P* < 0.05, ****P* < 0.005 vs. control, two-tailed student's *t* test; *n* = 4-5 mice per group for 2 independent experiments).

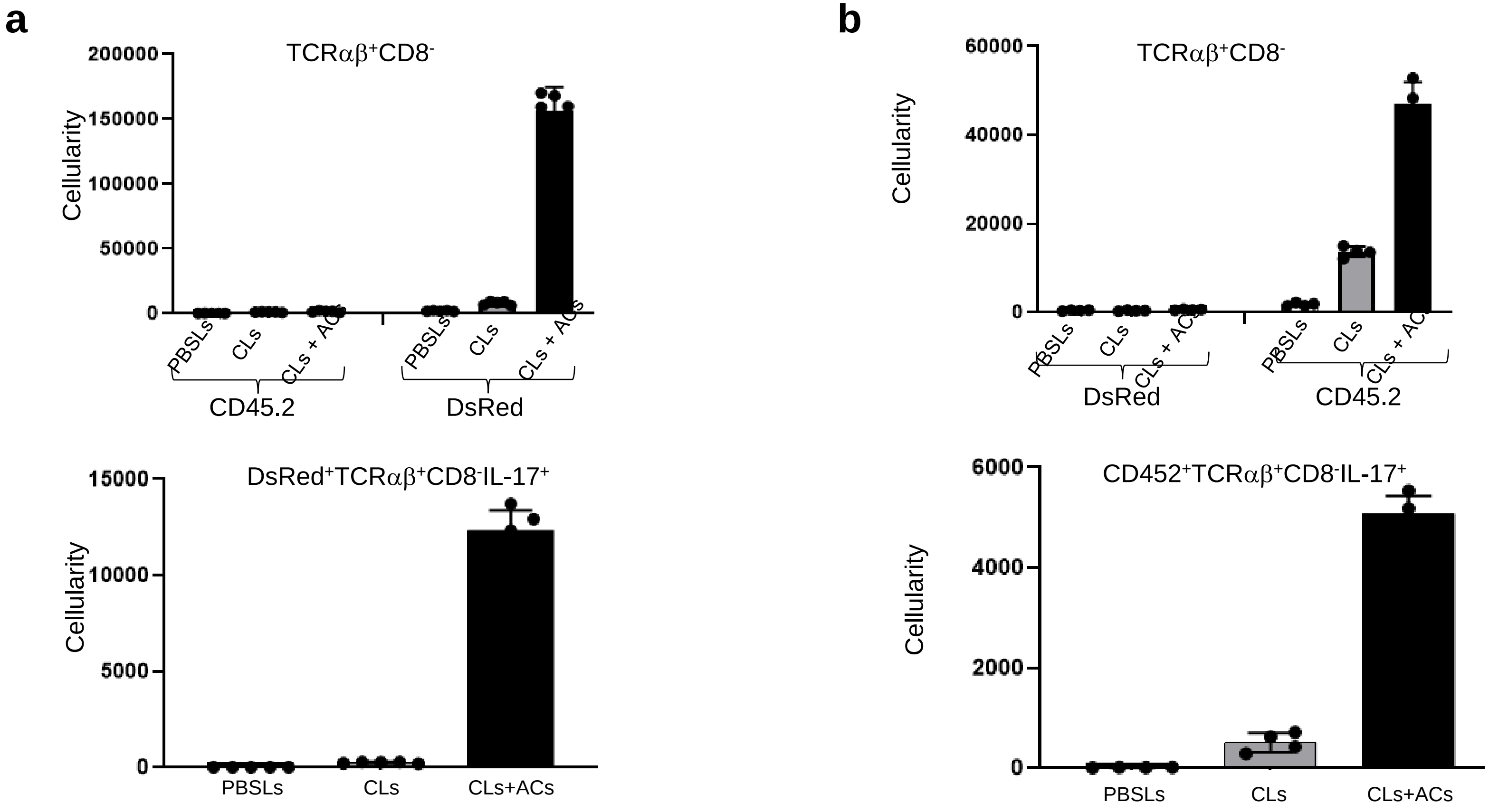

Supplementary Figure 6. Polyclonal CD8 T cells with a diverse repertoire from autoimmune mice acquired DN T cell like phenotype.

A mixed population of purified CD8 T cells ($TCRb^{+}NK1.1^{-}CD8^{+}$, 2 x 10^{6}/mouse, 1:1 ratio) from CD45.1 B6 mice and CD45.2 B6.*lpr.DsRed* (a) or from normal CD45.2 B6.*DsRed* mice and CD45.2 B6 *Aire*$^{-/-}$ mice (b) were transferred into either CD45.2 B6 (a) or CD45.1 B6 (b) recipients. 12 hrs later, recipients were administered control liposome, CLs, CLs plus apoptotic thymocytes prepared from B6 mice (2 x 10^{7}/mouse). Mice were euthanized after an additional 72 hrs. *n* = 5 mice per group for 2 independent experiments. Data represent the mean ± SEM.

(a) Upper: Bar graph shows the cellularity of $TCR\alpha\beta^{+}CD8^{-}$ T cells derived from transferred T cells in the spleens of CD45.2 B6 recipients with the indicated treatment. Low: Bar graph shows the cellularity of $DsRed^{+}TCR\alpha\beta^{+}CD8^{-}IL\text{-}17^{+}$ T cells derived from indicated donor in the spleens of CD45.2 B6 recipients with the indicated treatment.

(b) Upper: Bar graph shows the cellularity of $TCR\alpha\beta^{+}CD8^{-}$ T cells derived from transferred T cells in the spleens of CD45.1 B6 recipients with the indicated treatment. Low: Bar graph shows the cellularity of $CD45.2^{+}TCR\alpha\beta^{+}CD8^{-}IL\text{-}17^{+}$ T cells derived from indicated donor in the spleens of CD45.2 B6 recipients with the indicated treatment.

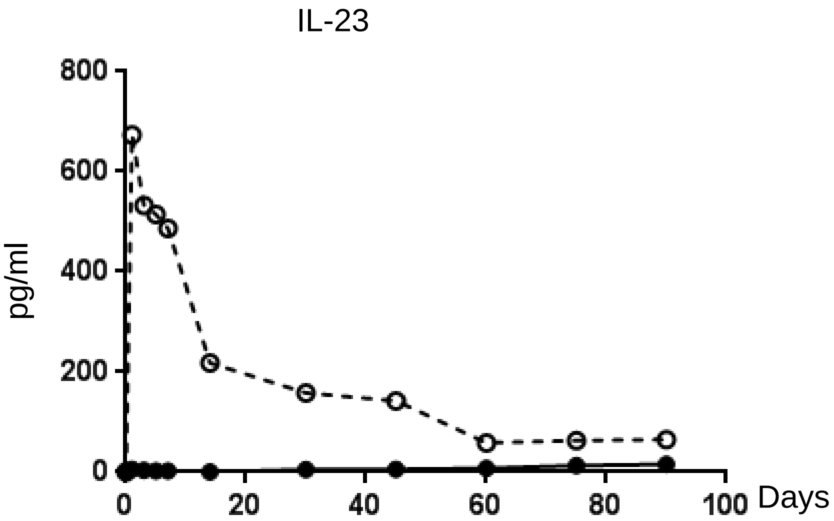

Supplementary Figure 7. Elevated IL-23 levels in the sera of mice subjected to IL-23 minicircle (MC) administration.

Eight week-old B6.*lpr* mice were injected i.v. with either GFP or IL-23 MC and euthanized 90 days after administration. GFP MC was applied as control. ELISA analysis of serum titers of IL-23 at the indicated time points after IL-23 minicircle administration. *n* = 5-7 mice per group in 2 independent experiments. Data represent the mean ± SEM (*n* = 5-6 mice per group for 2 independent experiments).

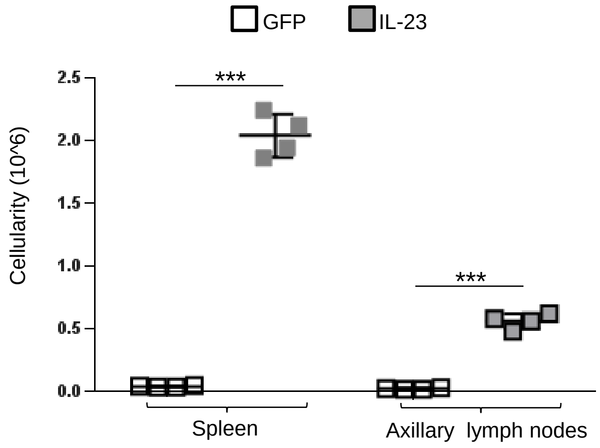

Supplementary Figure 8. Over-expression of IL-23 *in vivo* drives the expansion of DN T cells.

Eight week-old B6.*lpr* mice were injected i.v. with either GFP or IL-23 MC and euthanized 90 days after administration. Bar graph shows the cellularity of $CD3^{+}TCRb^{+}CD4^{-}CD8^{-}$ DN T cells in spleens or lymph nodes from mice with the indicated MC administration. Data represent the mean ± SEM. ****P* < 0.005 vs. control, two-tailed student's *t* test. *n* = 4 mice per group for 2 independent experiments.

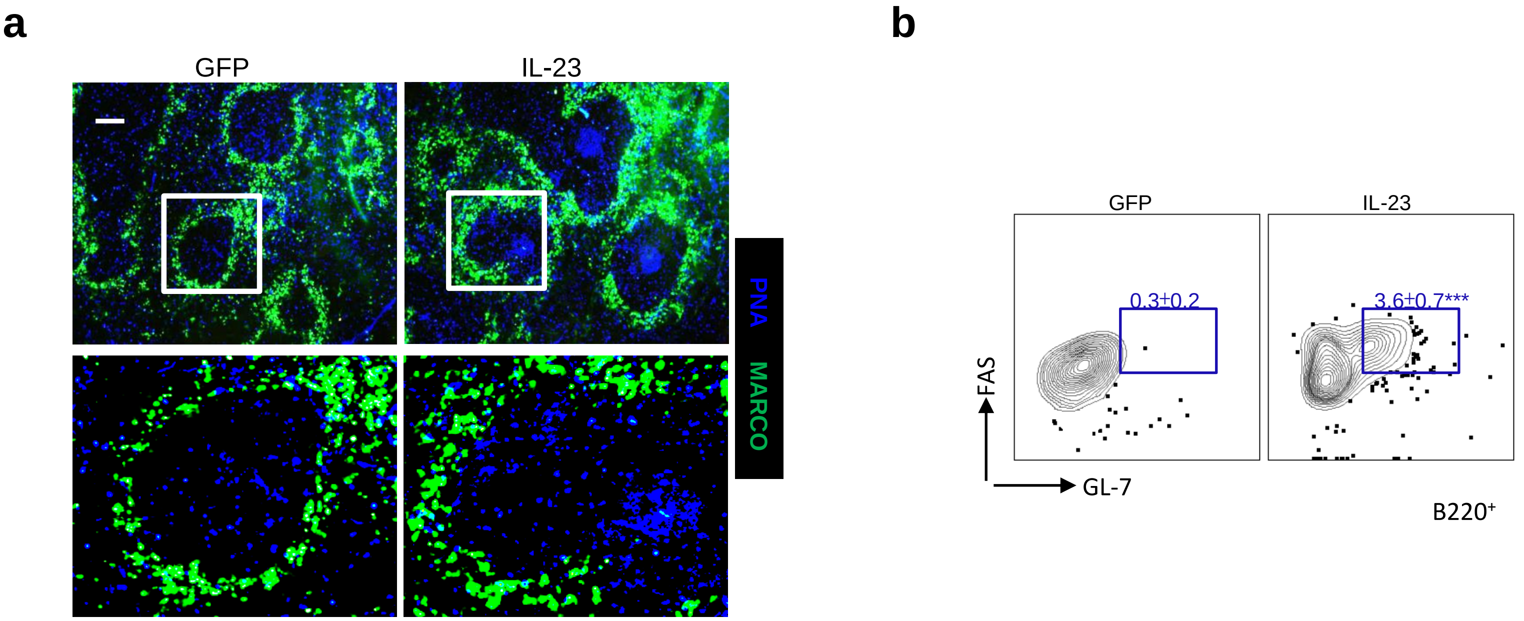

Supplementary Figure 9. Overexpression of IL-23 *in vivo* promotes germinal center B cell formation without interrupting MZM barrier integrity.

Eight week-old B6.*lpr* mice were injected i.v. with either GFP or IL-23 MC and euthanized 90 days after administration. (a) Representative immunofluorescent staining of MARCO^{+} marginal zone macrophages (Green) and PNA^{+} GC B cells (Blue) in the spleens from indicated mice. Upper: magnification, ×4. Scale bar: 200 um; Lower: Digitally magnified views of the boxed areas in the upper panels. (b) Flow cytometry quantitation of the percentage of GC B cells (CD19^{+}PNA^{+}FAS^{+}) in the spleens from indicated mice. Data represent the mean ± SEM (***P < 0.005 vs. control, two-tailed student's t test; n = 4 mice per group for 2 independent experiments).

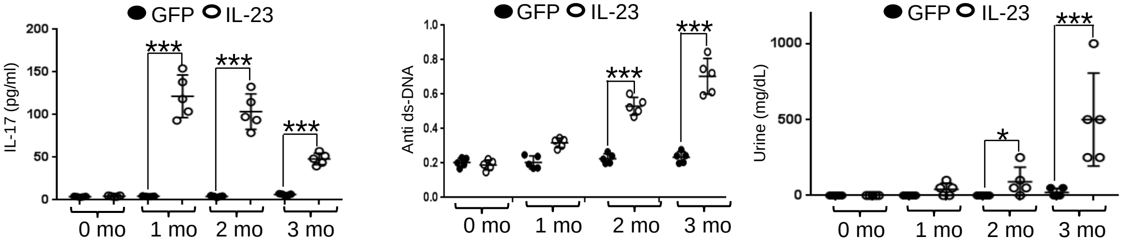

Supplementary Figure 10. Overexpression of IL-23 *in vivo* promotes inflammation and proteinuria.

Eight week-old B6.*lpr* mice were injected i.v. with either GFP or IL-23 MC and euthanized 90 days after administration. ELISA analysis of serum IL-17 (Left), serum anti-dsDNA IgG (Middle) and proteinuria (Right) from mice with indicated MC administration. Data represent the mean ± SEM (***P < 0.005 vs. control, two-tailed student's t test; n = 4 mice per group for 2 independent experiments).

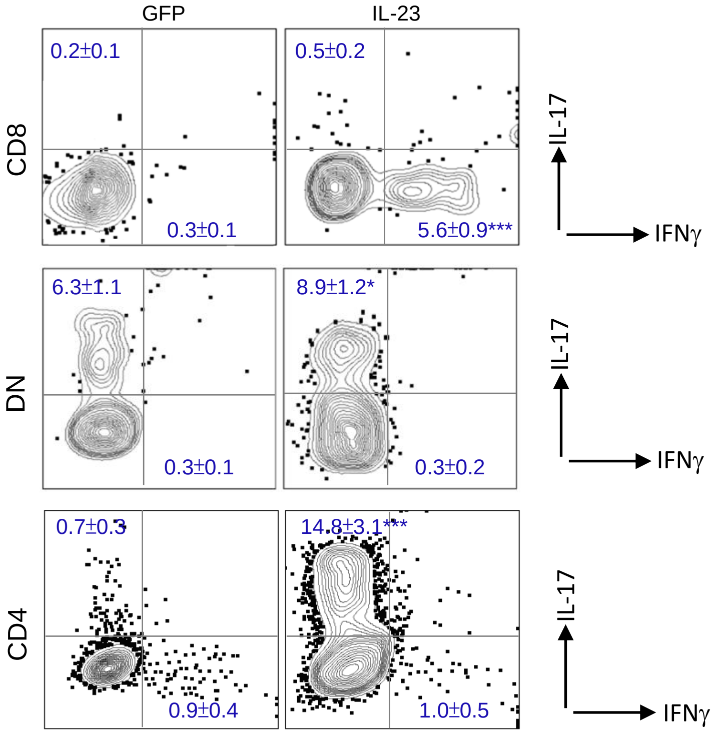

Supplementary Figure 11. Overexpression of IL-23 *in vivo* promotes IL-17 production in DN T cells.

Eight week-old B6.*lpr* mice were injected i.v. with either GFP or IL-23 MC and euthanized 90 days after administration. Flow cytometry quantitation of IL-17 and IFN-γ expression by splenic CD4, CD8 and DN T cells. Data represent the mean ± SEM (**P* < 0.05, ****P* < 0.005 vs. control, two-tailed student's *t* test; *n* = 4 mice per group for 2 independent experiments).

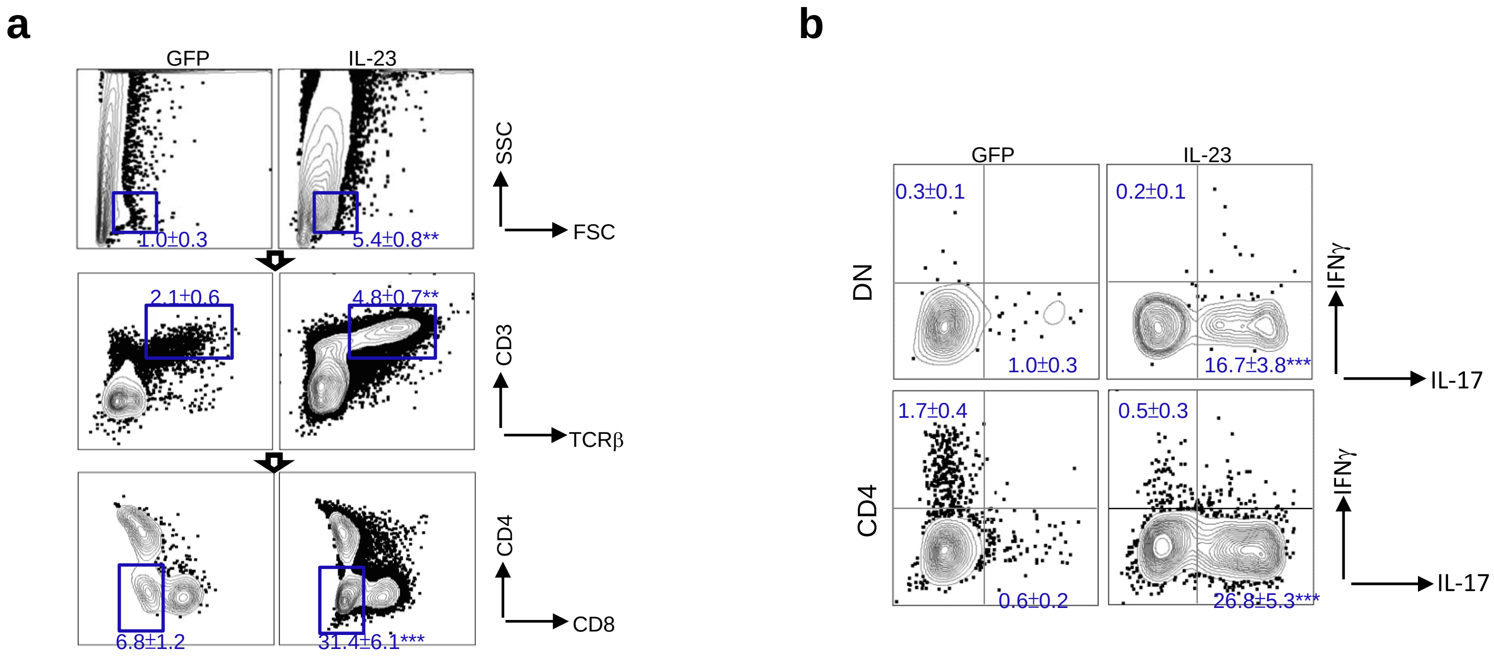

Supplementary Figure 12. Overexpression of IL-23 *in vivo* promotes intrarenal IL-17-producing DN T cells.

Eight week-old B6.*lpr* mice were injected i.v. with either GFP or IL-23 MC and euthanized 90 days after administration. (a) Flow cytometry analysis of infiltrating T cells in the kidneys from indicated mice. (b) Flow cytometry quantitation of IL-17 and IFN-γ production by intrarenal CD4 and DN T cells. Data represents the mean ± SEM (**P < 0.01, ***P < 0.005 vs. control, Student's t test; n = 4 mice per group for 2 independent experiments).

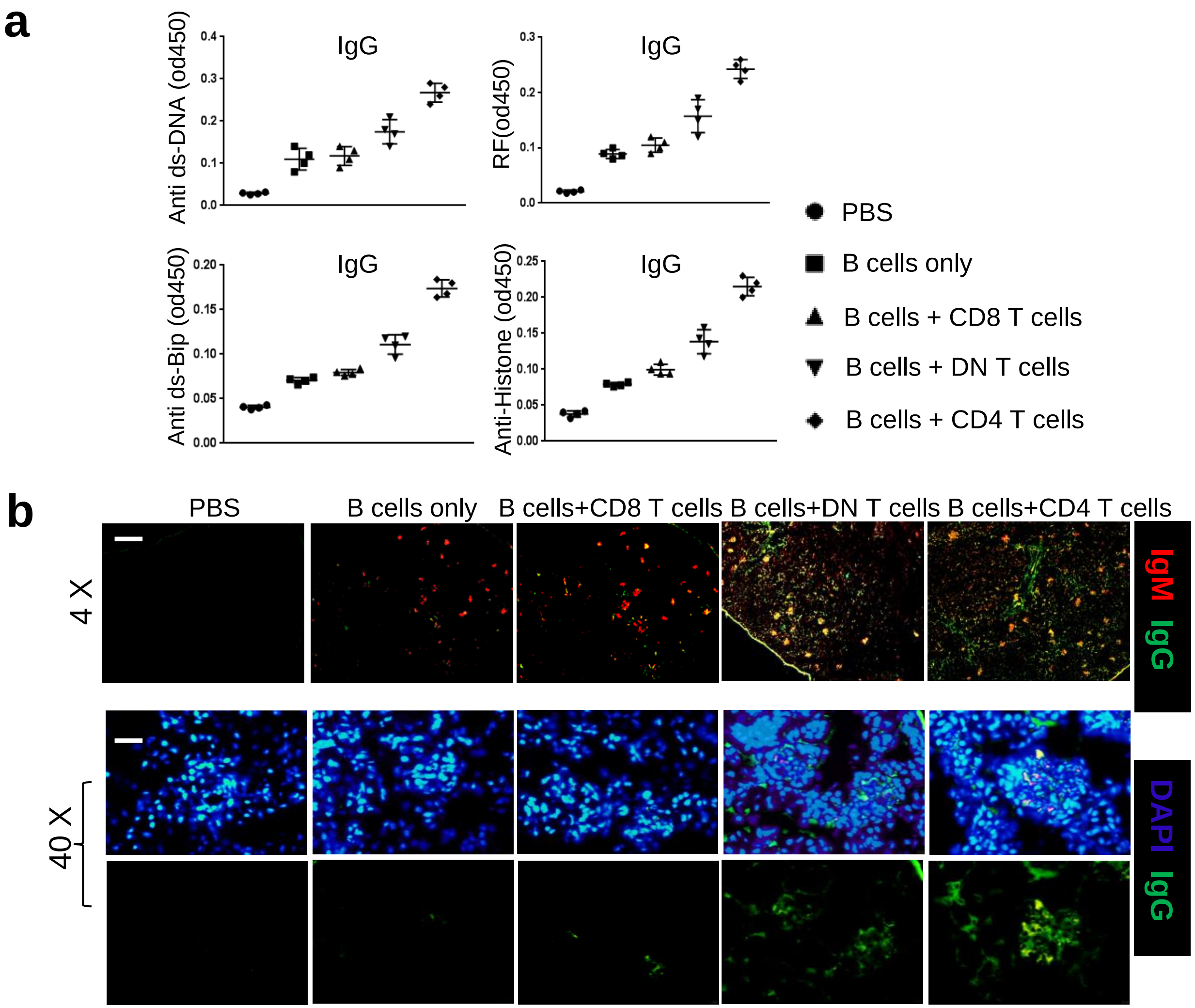

Supplementary Figure 13. DN T cells provide help to B cells to produce auto-antibody *in vivo*.

Spleen CD4, CD8 and DN T cells were enriched from IL-23 MC treated B6*lpr* mice and transferred into B6 Rag1$^{-/-}$ recipients which received purified B cells from 12 months old B6*lpr* mouse spleens one day before and mice were euthanized one month after cell transfer. *n* = 4 mice per group. Data represent the mean ± SEM.

(a) ELISA analysis of indicated auto-antibodies in the serum from indicated recipients.

(b) Immunohistochemistry staining of immune complex deposition in the kidneys from indicated recipients. Upper: magnification, ×4. Scale bar: 200 um; Lower: magnification, ×40. Scale bar: 20 um;

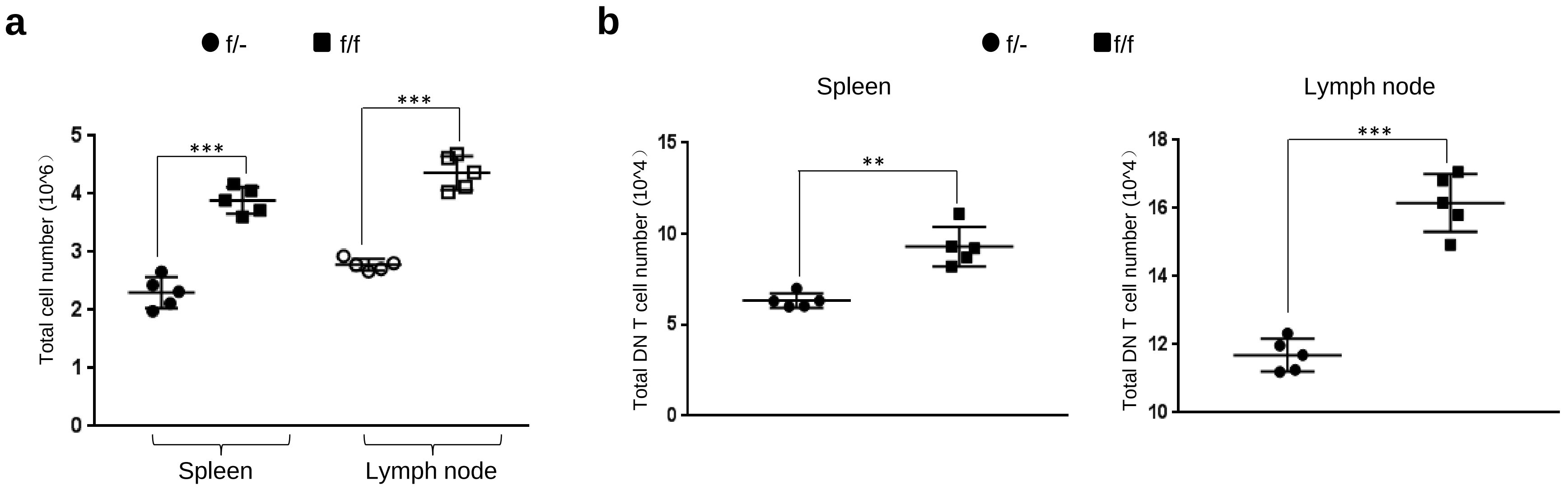

Supplementary Figure 14. Deficiency of *Tgfβ1* in macrophages promotes DN T cell development.

(a) Scatter plots show absolute number of total splenocytes from indicated mice. (b) Scatter plots show absolute number of DN T cells in spleens and lymph nodes from indicated mice. CD4 T cells: $CD3^{+}TCR\beta^{+}NK1.1^{-}CD4^{+}$, CD8 T cells: $CD3^{+}TCR\beta^{+}NK1.1^{-}CD8^{+}$, DN T cells: $CD3^{+}TCR\beta^{+}NK1.1^{-}CD4^{-}CD8^{-}$. Data represents the mean ± SEM (**$P < 0.01$, ***$P < 0.005$ vs. control, Student's *t* test; $n = 4$ mice per group for 2 independent experiments).

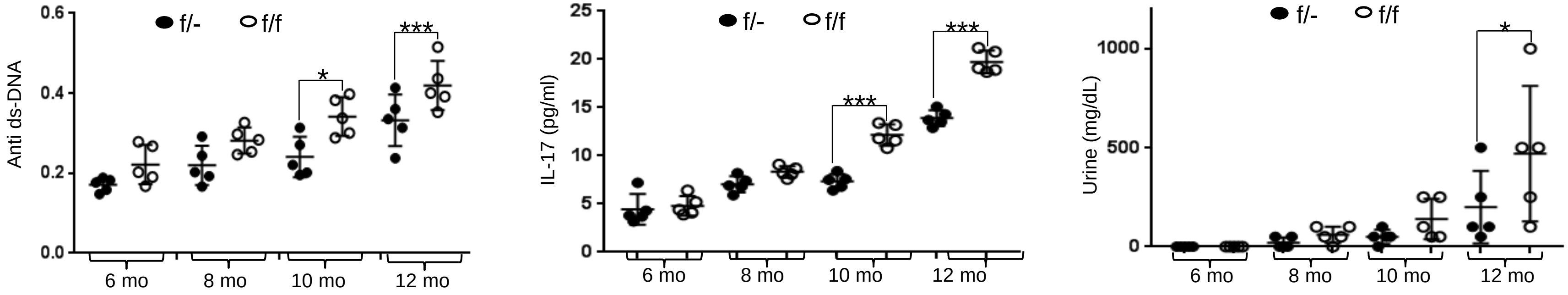

Supplementary Figure 15. Deficiency of *Tgfβ1* in macrophages promotes inflammation and proteinuria.

Scatter plots show the ELISA analysis of serum anti-dsDNA IgG (Left), serum IL-17 (Middle), and proteinuria (Right) from indicated mice. Data represent the mean ± SEM (***$P < 0.005$ vs. control, Student's t test; $n = 4$ mice per group for 2 independent experiments).

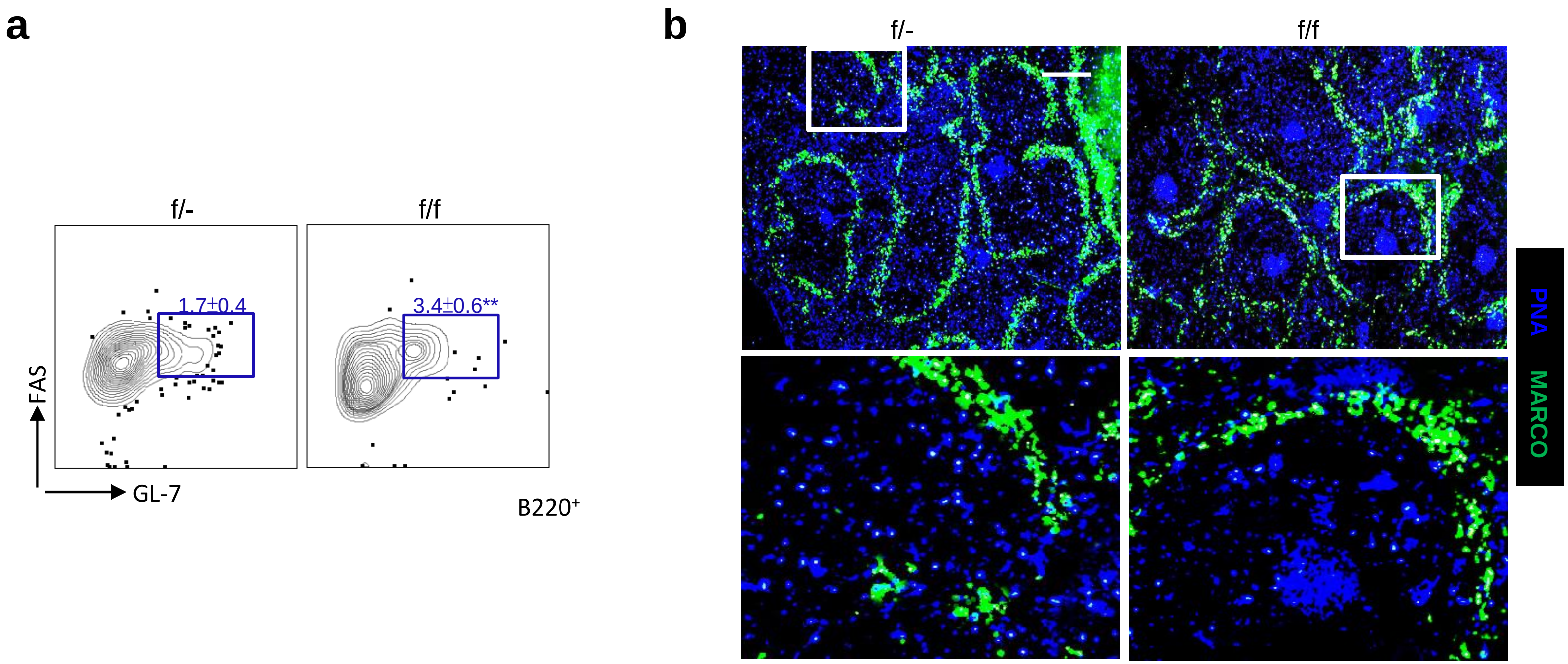

Supplementary Figure 16. Deficiency of *Tgfβ1* in macrophages promotes germinal center B cell formation without interrupting MZM barrier integrity.

Lyz2^{cre+}*Tgfβ1*$^{fl/fl}$ or *Lyz2*$^{cre+}$*Tgfβ1*$^{fl/+}$ B6.*lpr* mice at 12 months of age were applied. (a) Flow cytometry quantitation of the percentage of GC B cells (PNA^{+}FAS^{+}CD19^{+}) in the spleens from indicated mice. Data represents the mean ± SEM (**P < 0.01 vs. control, Student's t test; n = 4 mice per group for 2 independent experiments). (b) Representative immunofluorescent staining of MARCO^{+} marginal zone macrophages (Green) and PNA^{+} GC B cells (Blue) in the spleens from indicated mice. Upper: magnification, ×4. Scale bar: 200 um; Lower: Digitally magnified views of the boxed areas in the upper panels.

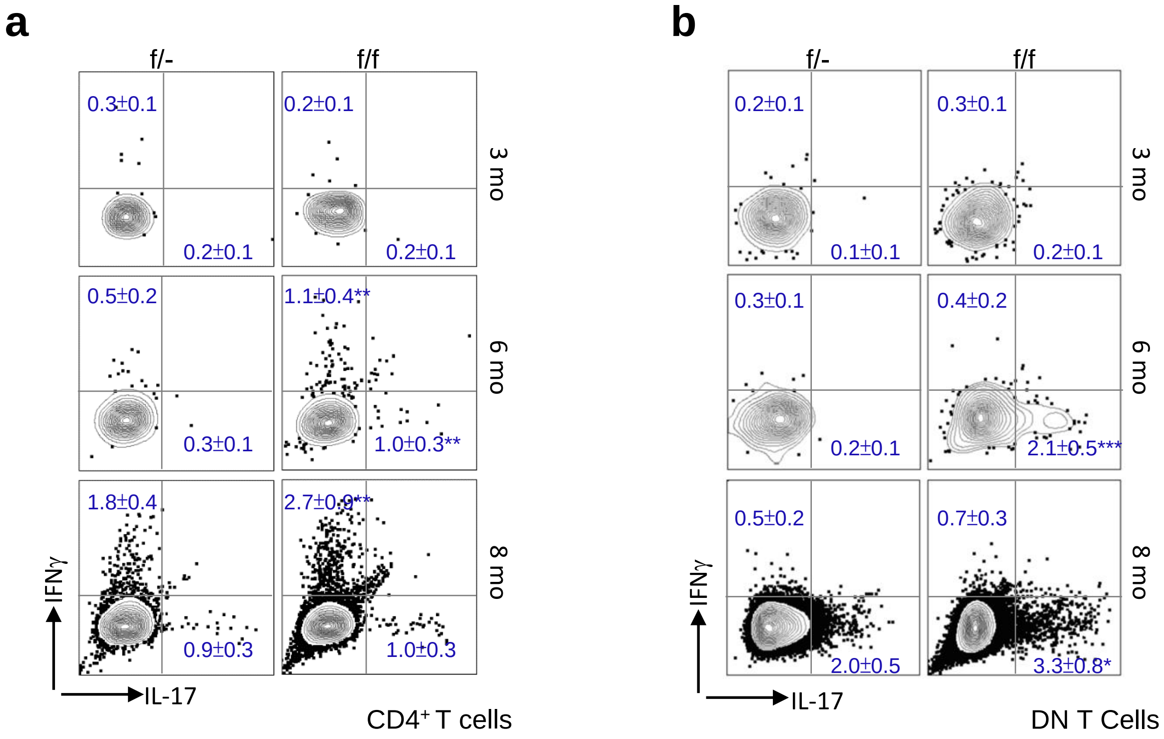

Supplementary Figure 17. Deficiency of *Tgfβ1* in macrophages promotes IL-17 production by DN T cells.

Flow cytometry quantitation of IL-17 and IFN-γ expression by splenic CD4 T cells (a) and DN T cells (b) from either *Lyz2*$^{cre+}$*Tgfβ1*$^{fl/fl}$ or *Lyz2*$^{cre+}$*Tgfβ1*$^{fl/+}$ B6.*lpr* mice with indicated ages. Data represents the mean ± SEM (**P* < 0.05, ***P* < 0.01, ****P* < 0.005 vs. control, Student's *t* test; *n* = 4 mice per group for 2 independent experiments).

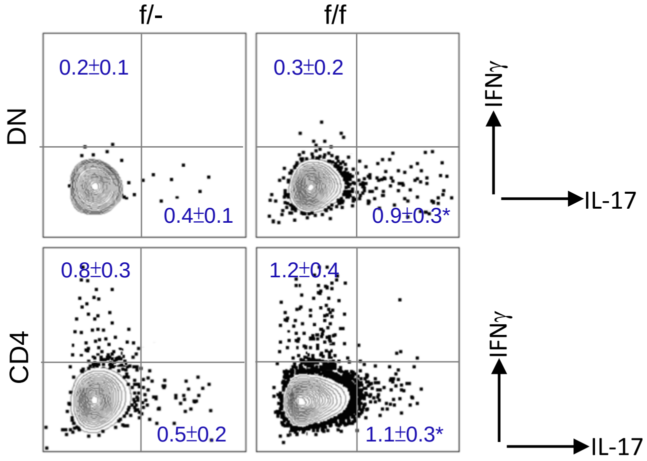

Supplementary Figure 18. Deficiency of *Tgfβ1* in macrophages promotes intrarenal IL-17 producing DN T cells.

Flow cytometry quantitation of IL-17 and IFN-γ expression by spleen DN T cells (Top) and CD4 T cells (Bottom) from either *Lyz2*$^{cre+}$*Tgfβ1*$^{fl/fl}$ or *Lyz2*$^{cre+}$*Tgfβ1*$^{fl/+}$ B6.*lpr* mice at 12 months of age. Data represents the mean ± SEM (**P* < 0.05, vs. control, Student's *t* test; *n* = 4 mice per group for 2 independent experiments).

	CD4	CD8	DN
**Clone counts**	133770	150976	194375
**Clone types**	44,891	81,313	42,026
**Counts of Top N clone types (1:10)**	5692	6274	10445
**Counts of Top N clone types (11:100)**	13125	7297	25005
**Proportion of Top N clone types (1:10)**	4.26%	4.16%	5.37%
**Proportion of Top N clone types (11:100)**	9.94%	4.83%	12.86%

Supplementary Table 1. TCRβ chain clonicity of spleen CD4, CD8 and DN T cells from a 16 week-old MRL.*lpr* mouse.

Spleen CD4, CD8 and DN T cells were FACS sorted from a 16 weeks old male MRL.*lpr* mouse and mRNAs were extracted from 1 million of each subset. The Library preparation and TCR profiling were performed using Takara SMARTer mouse TCRα/β profiling kit.

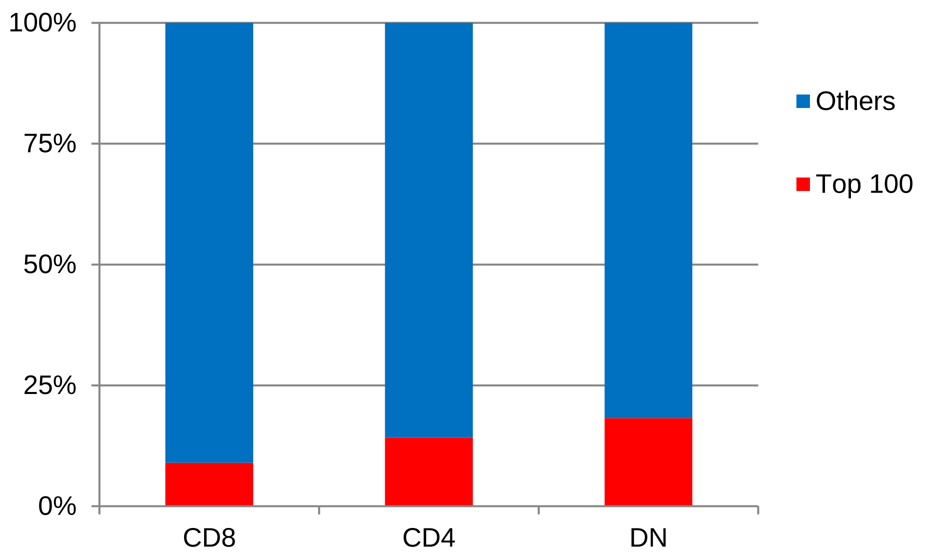

Supplementary Figure 19. Proportion of the top 100 clonetypes in indicated T cell subsets.

Bar graph shows quantitation of proportion of Top 100 clone types in spleen CD4, CD8 and DN T cells from a 16 week-old male MRL.*lpr* mouse.

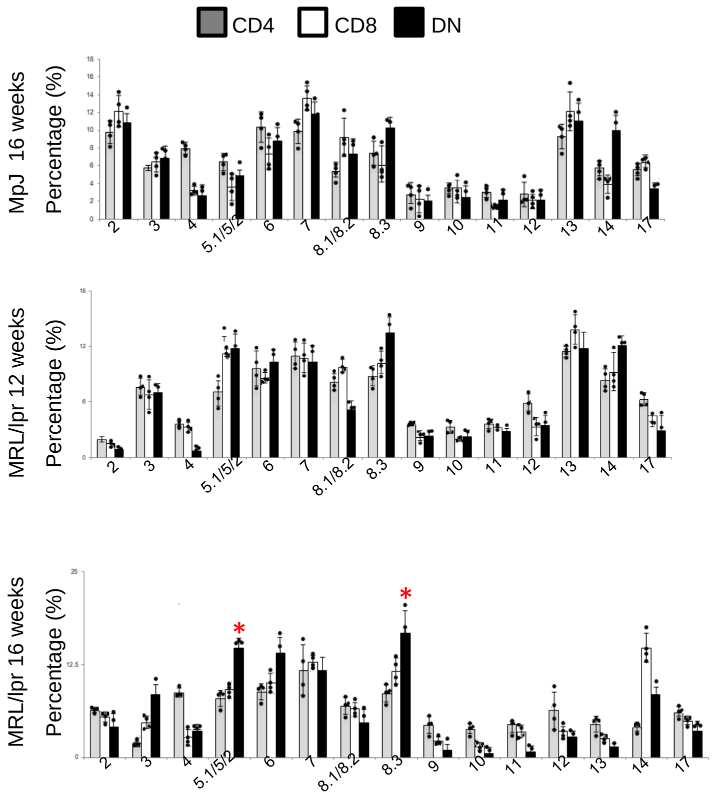

Supplementary Figure 20. Skewed Vβ repertoire of DN T cells.

Bar graphs show quantitation of TCR Vβ usage by CD4, CD8 and DN T cells of spleens from indicated mouse strains (gated in $CD3^{+}TCRb^{+}Ly49b^{-}$). Data represents the mean ± SEM (**P* < 0.05 vs. control, Student's *t* test; *n* = 4).

	CD8 T cells	DN cells
**Counts of Vβ5 clones**	7591	13374
**Vβ5 clone types**	4250	2891
**Proportion of Vβ5 clones**	5.03%	6.88%

Supplementary Table 2. Clonal expansion of Vβ5 in DN T cells.

CD8 and DN T cells were from a 16 week-old male MRL.*lpr* mouse spleen. The TCR profiling was performed using Takara SMARTer mouse TCRα/β profiling kit.

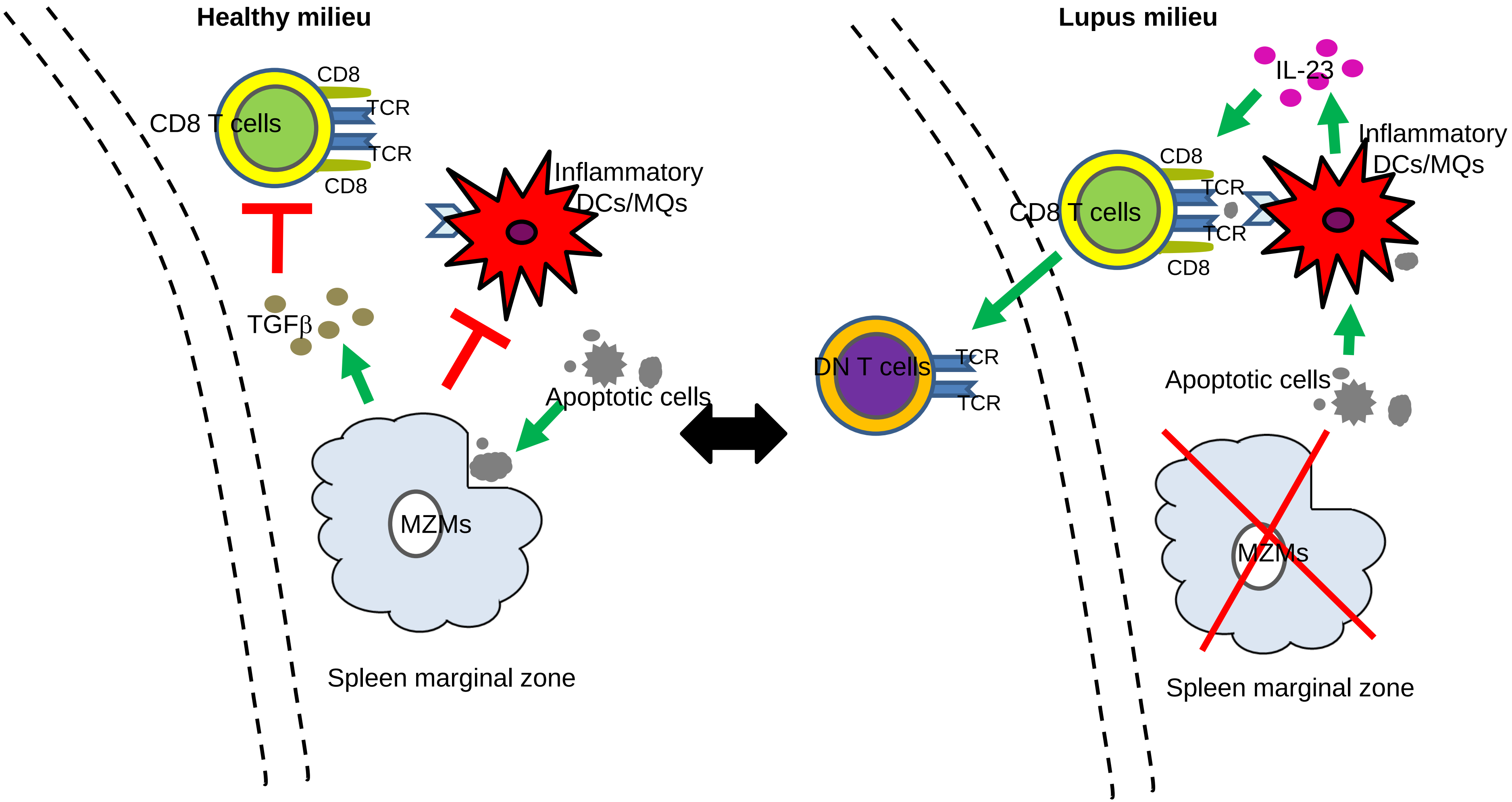

Supplementary Figure 21. Lupus milieu favors the conversion of self-reactive CD8 T cells to IL-17-producing double negative T cells.

Apoptotic cells (ACs) are normally taken up by MZMs and this process leads to release of tolerogenic cytokine TGFβ and reduction of AC autoantigens. In lupus, defective marginal zone barrier increases interactions of uncleared ACs with inflammatory dendritic cells or macrophages, which leads to production of inflammatory cytokines especially IL-23 with diminished TGFβ. In addition, defects in MZMs cause the accumulation of self-antigens derived from uncleared Acs, which activate self-reactive CD8 T cells which later acquired inflammatory DN T cell phenotypes.
